# Complement factor B polymorphism 32W protects against age-related macular degeneration

**Published:** 2011-04-20

**Authors:** Anne E. Hughes, Gemma M. Mullan, Declan T. Bradley

**Affiliations:** Centre for Public Health, Queen’s University Belfast, UK

## Abstract

**Purpose:**

The 32Q (rs641153; A) and 32W (rs12614; T) variants of complement factor B (CFB) cause less efficient complement activation in vitro than the common 32R variant. This is thought to be the reason that the 32Q variant is associated with decreased risk of age-related macular degeneration (AMD). We investigated whether the 32W variant was also associated with decreased risk of AMD.

**Methods:**

We genotyped 367 cases with neovascular AMD and 251 disease-free controls. Association with the disease phenotype was assessed by logistic regression for polymorphisms of *CFB* alone and in combination with smoking status and genetic risk markers of complement factor H (*CFH*) and HtrA serine peptidase 1 (*HTRA1*). We performed meta-analysis of all previously published reports of 32W allele frequency in AMD cases and controls.

**Results:**

The CFB variant 32W was associated with protection against neovascular AMD, compared to the common 32R variant (odds ratio 0.64, p<0.05, in logistic regression with CFB variants; odds ratio 0.53, p<0.05, in logistic regression with CFB variants, *CFH* haplotypes, *HTRA1* rs10490924 genotype, and smoking status). Meta-analysis (n=1,795) including this study and two others of neovascular AMD showed a combined odds ratio of 0.75 (p<0.05) for 32W, compared to 32R. Meta-analysis (n=2,600) of all reported studies of all types of AMD showed a combined odds ratio of 0.79 (p<0.01).

**Conclusions:**

Our study shows that the 32W variant of CFB is associated with protection against AMD, in keeping with evidence of its functional effect on the complement system. The protective effect is less strong than that associated with 32Q.

## Introduction

Age-related macular degeneration (AMD) is a major cause of visual impairment and blindness among older people [1]. The alternative pathway of the complement system, an ancient defense against infectious microbes [2,3], is implicated in the etiology and pathogenesis of the disease. Local and systemic complement activation occurs in individuals with AMD [4,5]. The proteins of the complement system and products of their activation are found to be raised in the circulation and in local deposits (drusen) in the retina [4]. Genome-wide association studies first revealed the association between polymorphisms in the complement factor H (*CFH*) gene and susceptibility to the disease [6-8]. Since then, risk-associated polymorphisms in complement factor I (*CFI*) [9], complement factor B (*CFB*) [10], complement component 3 (*C3*) [11], complement factor H-related 3 (*CFHR3*), and complement factor H-related 1 (*CFHR1*) [12] genes have been discovered and replicated. Progress has been made toward understanding the functional effects of the I62V and Y402H [13-15] polymorphisms in *CFH*, the deletion of *CFHR3* and *CFHR1* [16], and the R32Q polymorphism in *CFB* [17].

The central step of all three complement pathways is activation of the C3 molecule to C3b by a C3 convertase enzyme [3]. In the alternative pathway, the C3 convertase is made by the binding of C3b to CFB, forming the proenyzme C3bB. An enzyme, complement factor D (CFD), cleaves the CFB part of the proenzyme, separating the Ba fragment of the molecule from the active C3bBb complex. Montes et al. [17] studied the effect of two adjacent genetic polymorphisms on the function of human and recombinant CFB in vitro. These single nucleotide polymorphisms, rs641153 (R32Q) and rs12614 (R32W), affect the same codon and amino acid residue. Of the four possible combinations of bases, three are found to exist, with the combination of both major alleles resulting in an arginine (R), the minor allele of rs641153 with the major allele of rs12614 resulting in a glutamine (Q), and the minor allele of rs12614 with the major allele of rs641153 resulting in a tryptophan (W) at residue 32 (Table 1). This residue is in the Ba fragment of CFB, and is part of the binding site for C3b [17]. Compared to 32R, the 32Q variant of CFB binds with fourfold reduced affinity to C3b, reducing formation of the C3bB proenzyme and reducing lytic activity [17]. The 32W variant of CFB also exhibits reduced binding, proenzyme formation and lytic activity relative to 32R, but the reduction is less marked than that found with 32Q [17]. The 32Q variant is associated with a reduced risk of developing AMD [10]. The 32W variant has been studied by several groups [5,10,18,19], but has not been found to be associated with AMD risk. In this study, we investigate the risk of AMD associated with the 32R, 32W, and 32Q variants of CFB. Statistical analysis is presented for these variants alone, and in a logistic regression incorporating the other major genetic risk factors, *CFH* and *HTRA1*, and smoking status. We also perform a meta-analysis of data from all identified studies that have previously investigated rs12614 (R32W) in AMD.

**Table 1:** Complement factor B (CFB) codon 32 sequences and amino acids

**Codon 32 sequence**	**Amino acid**	**Symbol**
CGG	Arginine	R
TGG	Tryptophan	W
CAG	Glutamine	Q

Three sequence variants are found in codon 32 due to variations at the first base (rs12614: C/T) and second base (rs641153: A/G) of the codon. Three different amino acids are therefore found at residue 32 of the CFB protein. The genotype at both SNPs must be known to predict the presence of the 32R protein variant.

## Methods

### Study participants

The characteristics of the study group are shown in Table 2. Individuals with neovascular AMD in at least one eye were recruited between June 2002 and September 2006 from ophthalmology clinics in the Royal Victoria Hospital, Belfast, UK, the regional referral center for Northern Ireland. Diagnosis was made by clinical examination and fluorescein angiography. Stereoscopic digital fundus photographs were graded according to the Wisconsin age-related maculopathy grading system [20]. Cases had grade 4 neovascular AMD with or without geographic atrophy. Control participants were recruited from the local population, and they underwent retinal photography. Those included had grade 1a retinas (free of drusen or fewer than five hard drusen with a diameter of <63 µm). All participants self-reported European ancestry.

**Table 2:** Study characteristics.

**Characteristics**	**Grade 4 AMD cases**	**Grade 1a controls**
Participants (n)	369	251
Male	42%	48%
Female	58%	52%
Median age (range; years)	76 (54–93)	75 (68–92)

Age refers to the age at recruitment into the study.

The study conformed to the tenets of the declaration of Helsinki, and it was approved by the Office for Research Ethics Committees, Northern Ireland. All participants gave informed consent.

### Genotyping of rs641153 and rs12614

DNA was extracted from peripheral blood leucocytes or frozen buffy coat samples. We performed SNP genotyping of rs12614 and rs641153 by PCR (forward primer: 5′-ACA CAC CAT CCT GCC CCA G-3′; reverse primer: 5′-TAC CCC CTC CAG AGA GCA GG-3′) followed by DNA sequencing using the forward primer. All samples showing the minor allele of rs12614 were confirmed using SNaPshot (Applied Biosystems, Warrington, UK). Briefly, primer (5′-CAG GTG TGA CCA CCA CTC CAT GGT CTT TGG CC-3′) extension was performed on the PCR product using a fluorescent detection system. Data for the smoking status and *CFH* and *HTRA1* haplotypes of these participants were available from our previous studies [12,21].

### Statistical analysis

Deviation from the Hardy–Weinberg equilibrium was investigated by use of the exact method of Wigginton et al. [22] as used in PLINK v1.07 [23]. Logistic regression was performed using PASW v18. The numbers of copies (0, 1, or 2) of each *CFH* haplotype and *CFB* codon 32 variant carried by an individual were included as variables, omitting a reference haplotype from each gene. *CFH* haplotypes were tagged by rs6677604, rs3753396, rs419137, and rs2284664 (1: GACG; 2: GAAG; 3: GGAG; 4: GAAA; 5: AAAG), with haplotype 3 omitted as the reference. The heterozygous or homozygous state for the *HTRA1* risk allele (rs10490924; T) was included, with homozygosity for the major allele (rs10490924; G) omitted as the reference. Current smoking and past smoking were included in the model as binary variables, with having never smoked omitted as the reference. Associations between disease status and allele frequency in the studies performed by ourselves and other groups were investigated using Pearson’s χ^2^ test in Epi-Info v6. To enable comparison of data sets from other groups, we used their published data, multiplying allele frequencies by the number of cases or controls to give the allele count if these were not published. For the meta-analysis, we compared the counts of 32W and 32Q alleles to the reference variant, 32R. Statistical significance was accepted at p<0.05 for all tests.

## Results

Both rs641153 and rs12614 were in Hardy–Weinberg equilibrium in cases and controls. The genotyping rate was 99.7%. *CFB* genotype data were available for 618 participants.

### Logistic regression of complement factor B 32R, 32W, and 32Q

The 32Q and 32W variants of CFB were found to be protective against the disease when compared to the 32R variant (Table 3). The 32Q variant was associated with an odds ratio of 0.43 (p=8.1×10^−5^), and the 32W variant was associated with an odds ratio of 0.64 (p=0.04).

**Table 3:** Logistic regression of complement factor B (CFB) variants.

**Variant**	**Odds ratio**	**95% Confidence interval**		**p value**
		**Lower**	**Upper**	
32R	1 (reference)			
32W	0.64	0.42	0.98	0.04
32Q	0.43	0.28	0.65	8.1×10^−5^
Constant	1.81			2.0×10^−9^

A logistic regression of the three amino acid variants at residue 32 of the CFB protein. The genotypes were coded as 0, 1 or 2 copies of each variant.

**Table 4:** Logistic regression of complement factor B (CFB) codon 32 type, complement factor H (*CFH*) haplotypes, *HTRA1* rs10490924 genotype and smoking status.

**Covariates**	**Odds ratio**	**95% Confidence interval**		**P value**
		**Lower**	**Upper**	
*CFH* haplotype 1	1.91	1.20	3.04	6.7×10^−3^
*CFH* haplotype 2	2.41	1.64	3.52	6.5×10^−6^
*CFH* haplotype 4	1.12	0.71	1.75	0.63
*CFH* haplotype 5	0.47	0.28	0.78	4.0×10^−3^
CFB 32W	0.53	0.31	0.91	0.02
CFB 32Q	0.33	0.19	0.56	5.1×10^−5^
*HTRA1* risk heterozygote (AT)	3.40	2.20	5.26	3.9×10^−8^
*HTRA1* risk homozygote (TT)	25.04	11.24	55.78	3.3×10^−15^
Ex-smoker	1.73	1.10	2.71	0.02
Current smoker	3.61	2.00	6.49	1.9×10^−5^
Regression constant	0.29			5.9×10^−5^

A logistic regression of genetic variants and smoking status. *CFB* and *CFH* data were coded as 0, 1 or 2 copies of each allele. Complete data were available for 351 cases and 222 controls to allow inclusion in the logistic regression model. *CFH* haplotypes are defined in Methods.

**Table 5:** Review and meta-analysis of all identified previous reports of rs12614 (R32W) polymorphism in AMD

**Study**	**Case phenol** **type**	**Population**	**Study size**	**Cases**			**Controls**			**32W**			**32Q**		
				32W	32Q	32R	32W	32Q	32R	OR	95%CI	P	OR	95%CI	P
Current study	NV	European	618	52	39	643	49	58	395	0.65	0.42–1.00	0.04	0.41	0.26–0.64	2.9x10^−05^
[10]	NV	European-American	548	52	21	473	55	61	434	0.87	0.57–1.32	0.49	0.32	0.18–0.54	4.2 x10^−06^
[18]	NV	Caucasian	629	70	37	731	41	48	330	0.77	0.50–1.18	0.21	0.35	0.22–0.56	1.7 x10^−06^
[10]	GA	European-American	366	20	4	158	55	61	434	1.00	0.56–1.77	1.00	0.18	0.05–0.53	2.7 x10^−04^
[10]	Early	European-American	459	37	19	312	55	61	434	0.94	0.59–1.49	0.77	0.43	0.24–0.76	1.7 x10^−03^
[10]	NV, GA, Early	European-American	823	109	44	943	55	61	434	0.91	0.64–1.31	0.60	0.33	0.22–0.51	2.6 x10^−08^
[19]	NV, GA	Indian	351	52	27	274	55	90	204	0.70	0.45–1.09	0.10	0.22	0.14–0.36	3.1 x10^−11^
[5]	NV, GA, Drusen	Caucasian	179	25	6	193	7	10	117	2.17	0.86–5.69	0.08	0.36	0.11–1.12	0.05
Meta-analysis	NV		1795	174	97	1847	145	167	1159	0.75	0.59–0.96	0.02	0.36	0.28–0.47	4.6 x10^−15^
Meta-analysis	All		2600	308	153	2784	207	267	1480	0.79	0.65–0.96	0.01	0.30	0.25–0.38	1.8 x10^−31^

p values calculated using Pearson’s χ^2^ test and allelic model. The odds ratios and p values for 32W and 32Q are calculated with reference to 32R. Phenotype and population descriptions are as reported in the original papers. NV=Neovascular AMD; GA=Geographic Atrophy; OR=Odds Ratio; CI=Confidence Interval; p=p value. The numbers from the Spencer et al. [18] and Kaur et al. [19] papers are derived from reported percentages and frequencies, therefore may be slightly inaccurate.

### Logistic regression incorporating smoking status and genotypes in complement factor B, complement factor H, and *HTRA1*

The 32Q and 32W variants of CFB were found to be protective against AMD when smoking status and other known genetic risk loci were integrated into a logistic regression (Table 4). The gradation of effect was the same as in the individual regression, with 32Q being more protective than 32W (odds ratios 0.33 and 0.53, respectively). *CFH* haplotypes 1 and 2, which carry the Y402H risk allele [12], were associated with increased risk relative to haplotype 3 (odds ratios 1.91 and 2.41, respectively). *CFH* haplotype 5, which carries the deletion of *CFHR3* and *CFHR1* [12], was protective against AMD (odds ratio 0.47). The *HTRA1* risk allele was associated with increased risk, particularly in homozygosity (odds ratio 25.04). Past and current smoking were associated with increased risk (odds ratios 1.73 and 3.61, respectively) compared to having never smoked.

### Meta-analysis of published studies of complement factor B 32R, 32W, and 32Q

The 32Q variant of CFB was significantly associated with protection from AMD in all of the studies that also reported data for 32W (Table 5). The 32W variant was associated with moderate protection from neovascular AMD in the current study and the Gold [10] and Spencer [18] studies, though the finding was not statistically significant in the latter two studies. Meta-analysis of these three neovascular AMD studies showed an odds ratio of 0.75 (p=0.02) for the 32W variant, compared to the 32R variant. Gold et al. [10] reported data for other AMD phenotypes, while Kaur [19] and Scholl [5] reported studies of mixed phenotypes. The trend in these studies was less consistent, with Gold reporting no association for geographic atrophy (though with wide confidence intervals) [10] and Scholl reporting a nonsignificant increase in risk (in a small study of mixed phenotype, with wide confidence intervals) [5]. Meta-analysis of all the studies that included all phenotypes showed that the 32W variant of CFB was associated with a significant reduction in risk of AMD (odds ratio 0.79, p=0.01).

## Discussion

Significant association between the minor allele at rs12614 in *CFB* (32W) and decreased risk of neovascular AMD has not been reported previously. The study by Montes et al. [17] provided evidence that this polymorphism was associated with less efficient alternative complement pathway activation due to weakened binding between CFB and C3b. Their study also showed that the established protective 32Q variant (associated with the minor allele at rs641153) was associated with a more extreme reduction in complement activation relative to 32R. We investigated the hypothesis that the genetic variant causing 32W was associated with protection from AMD, and found this to be the case when compared to 32R by Pearson’s χ^2^, by logistic regression with rs641153 and by logistic regression that included other known risk factors for AMD. The two other studies of neovascular AMD (Gold [10] and Spencer [18]) showed nonsignificant protective effects (odds ratios 0.87 and 0.77, respectively). When all neovascular cases were examined in a meta-analysis, a statistically significant, moderately protective odds ratio (0.75; p=0.02) was revealed. A more significant, less strong odds ratio (0.79; p=0.01) was revealed when meta-analysis of all AMD cases was undertaken.

This association has not been detected previously. This discord may reflect chance findings of either our study or the studies of others. However, combined analysis of all available data supports the conclusion that CFB 32W is associated with moderate protection against neovascular AMD, a finding that is in keeping with the study by Montes et al., which showed decreased complement activation relative to CFB 32R [17].

Our study provides further evidence that an individual’s risk of AMD is affected by gene polymorphisms that affect the function and interaction of the alternative complement pathway proteins.
